# Potential Coronaviral Inhibitors of the Nucleocapsid Protein Identified In Silico and In Vitro from a Large Natural Product Library

**DOI:** 10.3390/ph15091046

**Published:** 2022-08-24

**Authors:** Alexandra Pohler, Sara Abdelfatah, Max Riedl, Christian Meesters, Andreas Hildebrandt, Thomas Efferth

**Affiliations:** 1 Department of Pharmaceutical Biology, Institute of Pharmaceutical and Biomedical Sciences, Johannes Gutenberg University, Staudinger Weg 5, 55128 Mainz, Germany; 2 High Performance Computing Group, University of Mainz, 55131 Mainz, Germany; 3 Institute for Computer Science, University of Mainz, 55131 Mainz, Germany

**Keywords:** COVID-19, drug discovery, microscale thermophoresis, natural products, nucleocapsid protein, virtual drug screening, SARS-CoV-2

## Abstract

The nucleocapsid protein (NP) is one of the main proteins out of four structural proteins of coronaviruses including the severe acute respiratory syndrome coronavirus 2, SARS-CoV-2, discovered in 2019. NP packages the viral RNA during virus assembly and is, therefore, indispensable for virus reproduction. NP consists of two domains, i.e., the N- and C-terminal domains. RNA-binding is mainly performed by a binding pocket within the N-terminal domain (NTD). NP represents an important target for drug discovery to treat COVID-19. In this project, we used the Vina LC virtual drug screening software and a ZINC-based database with 210,541 natural and naturally derived compounds that specifically target the binding pocket of NTD of NP. Our aim was to identify coronaviral inhibitors that target NP not only of SARS-CoV-2 but also of other diverse human pathogenic coronaviruses. Virtual drug screening and molecular docking procedures resulted in 73 candidate compounds with a binding affinity below −9 kcal/mol with NP NTD of SARS-CoV-1, SARS-CoV-2, MERS-CoV, HCoV-OC43, HCoV-NL63, HoC-229E, and HCoV-HKU1. The top five compounds that met the applied drug-likeness criteria were then tested for their binding in vitro to the NTD of the full-length recombinant NP proteins using microscale thermophoresis. Compounds (**1**), (**2**), and (**4**), which belong to the same scaffold family of 4-oxo-substituted-6-[2-(4a-hydroxy-decahydroisoquinolin-2-yl)2H-chromen-2-ones and which are derivates of coumarin, were bound with good affinity to NP. Compounds (**1**) and (**4**) were bound to the full-length NP of SARS-CoV-2 (aa 1–419) with Kd values of 0.798 (±0.02) µM and 8.07 (±0.36) µM, respectively. Then, these coumarin derivatives were tested with the SARS-CoV-2 NP NTD (aa 48–174). Compounds (**1**) and (**4**) revealed Kd-values of 0.95 (±0.32) µM and 7.77 (±6.39) µM, respectively. Compounds (**1**) and (**4**) caused low toxicity in human A549 and MRC-5 cell lines. These compounds may represent possible drug candidates, which need further optimization to be used against COVID-19 and other coronaviral infections.

## 1. Introduction

In Wuhan, the capital of Hubei Province, China, various cases of unknown severe lung disease were reported for the first time in December 2019 that were caused by a novel virus, termed the severe acute respiratory syndrome coronavirus (SARS-CoV-2). The disease was called coronavirus disease 2019 (COVID-19). Since March 2020, COVID-19 has been declared a global pandemic by the World Health Organization (WHO) [1]. Through easy human-to-human transmission via small droplets and aerosols, the virus was distributed very quickly all over the world. The symptoms range widely between symptomless (mild), moderate, and severe pneumonia, acute respiratory distress symptom, and death [2]. Previously, in the years 2002/2003, the outbreak of another coronavirus (SARS-CoV-1) in Guangdong Province, China, affected 29 countries. The death rate of SARS-CoV-1 was about 10% [3,4]. In 2012–2014, the Middle East respiratory syndrome-related coronavirus (MERS-CoV) appeared in Saudi Arabia. Yet, the death rate was about 35% [5]. There are also four coronaviruses that cause the common cold in humans: HCoV-299E, HCoV-OC43, HCoV-NL63, and HCoV-HKU1 [6]. By contrast, SARS-CoV-1, SARS-CoV-2, and MERS-CoV cause severe partwise lethal illnesses [7,8,9]. This history of coronavirus transmission to humans indicates that this virus family could be a source of further serious pandemics in the future.

SARS-CoV-2 contains an unusually large genome with 29.8 kb of (+) single-stranded RNA. Next to the coded structural proteins such as spike (S), membrane (M), and envelope (E), the coronavirus expresses a nucleocapsid protein (N or NP) [10]. After infection, NP is the most abundantly expressed protein [11]. It contributes to the virus replication and assembly by binding and organizing the viral RNA into a ribonucleoprotein [12,13]. It contains 419 residues and consists of two domains, the N-terminal domain (NTD) and the C-terminal domain (CTD). Both domains are interconnected to each other by a phosphorylatable linker region (LR) [14]. The NTD binds the viral RNA, whereas the CTD both binds the RNA and dimerizes NPs. The LR contributes to these activities [15]. NP interacts with different signaling pathways in human cells and modulates some of them, favoring viral expression. The translation machinery, stress response, and inflammatory response of the host are influenced and upregulated by NP signaling [16,17,18,19]. NP also interacts with genes of the inflammasome, e.g., gasdermin D [20], NLRP3, and related pathways. Moreover, NP upregulates NF-κB and subsequently the interleukin expression that promotes the inflammation process and contributes to the so-called “cytokine storm” [21] and a continuation of the disease termed “long-COVID”.

Since the start of the pandemic, a series of mutational phenotypes occurred in the spike protein of SARS-CoV-2. Among them, the ‘variants of concern’ (VOC) bear a high potential for increased virus transmissibility. They also alter the viral antigenicity properties. This is of high importance as it may lead to SARS-CoV-2 variants that escape immune recognition and immune response upon vaccination [22,23]. It can be expected that viral evolution will continue, resulting in further new variants with increased infectivity, transmissibility, virulence, and decreased antigenicity. This represents a considerable challenge for the prevention of COVID-19 by vaccination and treatment by chemical drugs [22,24,25]. Although large portions of the population have been vaccinated against SARS-CoV-2 in industrialized countries, as yet pharmacological treatment of the disease remains a tremendous obstacle. The neutralizing activity of plasma from vaccinated individuals was significantly less against VOC mutations [26]. Likewise, the available vaccinations reveal differing effectiveness between wildtype SARS-CoV-2 and VOC phenotypes [27], and chemical drugs with approved activity against variants with wildtype or mutated spike proteins are missing as of yet.

Therefore, we hypothesize that the identification of small molecules that are selectively active against a druggable target different from the spike protein might be attractive because such a compound may address all spike phenotypes independent from their mutational status. Furthermore, several computational simulation models have predicted that the probability is quite high for the next virus epidemic/pandemic to come; it may be wise to have candidate drugs at hand that are not only active against wildtype and mutated SARS-CoV-2, but also against other coronaviruses and eventually also against still unknown coronaviruses that might appear in the future. These molecules could bind to the target of interest that harbors lower mutations across different strains. Some targets are conserved among the coronavirus family members.

In this study, we focused on small molecules targeting the RNA binding domain of NP in all seven human pathogenic coronaviruses in an attempt to identify inhibitors with broad spectrum activity.

## 2. Results

### 2.1. Multiple Sequence Alignment

**Figure 1:**
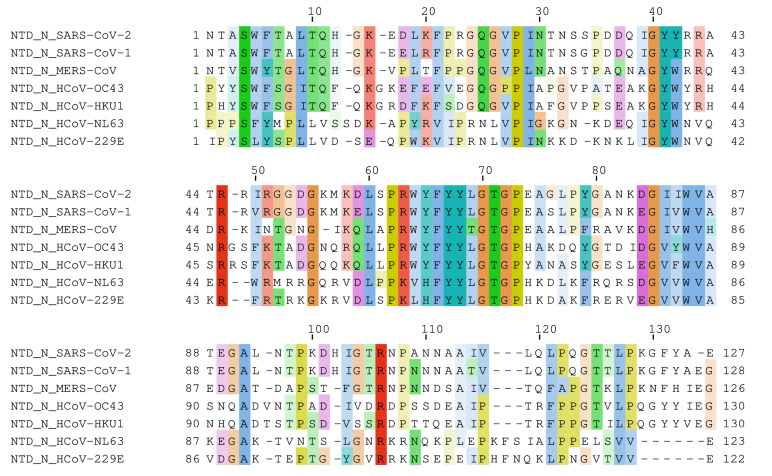
Multiple sequence alignment between the NTD of the nucleocapsid proteins of 7 different coronaviruses, i.e., SARS-CoV-2 (PDB: P0DTC9), SARS-CoV-1 (PDB: P59595), MERS-CoV (I PDB: K9N4V7), HCoV-OC43 (PDB: P33469), HCoV-NL63 (PDB: Q6Q1R8), HCoV-229E (PDB: P15130), and HCoV-HKU1 (PDB: Q5MQC6).

We performed a multiple sequence alignment for the NP NTD domains of all human pathogenic coronaviruses, i.e., SARS-CoV-2, SARS-CoV-1, MERS-CoV, HCoV-OC43, HCoV-HKU1, HCoV-NL63, and HCoV-229E. As shown in Figure 1, there are multiple amino acids shared across the different NTD sequences. The color code shows the different properties of the amino acids, e.g., positive charged amino acids were represented in red, negative charged amino acids in magenta, hydrophobic amino acids in blue, polar amino acids in green, cysteines in pink, glycines in orange, prolines in yellow, aromatic amino acids in cyan, and non-conserved amino acids in white. The amino acids Ser4, Pro28, Gly40, Tyr41, Arg47, Gly55, Leu60, Pro62, Phe66, Tyr67, Tyr68, Gly70, Thr71, Gly72, Pro73, Gly86, Trp88, Val89, Ala91, and Arg106 were represented in all NP NTDs. It can be assumed that conserved amino acids are important for the function of the proteins. Therefore, we considered these residues for the subsequent drug screening steps.

We performed a homology analysis of the seven coronaviral NTD sequences taken from UniProt.kb using Clustal Omega. The highest homology was found between the NTDs of SARS-CoV-2 and SARS-CoV-1 with 92.54% similarity while the NTD of MERS-CoV resulted in 60% similarity to SARS-CoV-2 followed by the NTDs of HCoV-HKU1, HCoV-OC43, HCoV-229E, and HCoV-NL63 with homologies of 44.09%, 41.73%, 33.90%, and 31.09%, respectively.

### 2.2. Literature Research for Known Active Residues in RNA Binding of the NTD

The essential residues for RNA binding of the NP NTD from SARS-CoV-2, SARS-CoV-1, MERS-CoV, HCoV-OC43, and HCoV-NL63 were reported in the literature (Table 1). The amino acids that are uniformly mentioned for RNA-binding in more than one NTD sequence are underlined. In almost every NTD, Arg45, Tyr62, Tyr64, Arg102 (referred to SARS-CoV-2 NTD) were involved in the binding function.

### 2.3. Virtual Drug Screening and Molecular Docking

**Figure 2:**
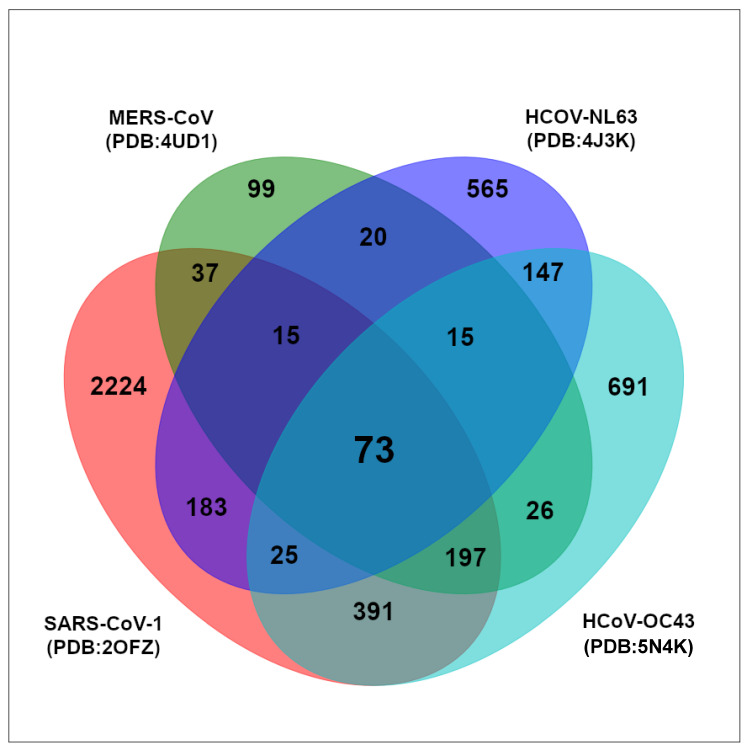
Venn diagram of the top 30% natural products bound to SARS-CoV-2 NP NTD (PDB: 6M3M) and re-screening-results to NP NTD of four other coronaviruses SARS-CoV-1 (PDB:2OFZ), MERS-CoV (PDB:4UD1), HCoV-OC43 (PDB:4J3K), and HCoV-NL63 (PDB:5N4K).

By implementing bioinformatic methods, we performed a virtual drug screening using a ZINC-based natural product library of more than 210,000 compounds and the NTD domain of SARS-CoV-2 NP. We used the Vina LC software to run an established workflow on a high-performance computer (MOGON). The protein binding pocket was determined based on amino acids known to be involved in the RNA-binding of the NP NTD. As a first step of the screening, SARS-CoV-2 NP NTD (PDB:6M3M) was used as target protein. The top 30% of compounds with the lowest binding energies were subsequently rescreened with the NTDs of SARS-CoV-1 (PDB:2OFZ), MERS-CoV (PDB:4UD1), HCoV-OC43 (PDB:4J3K), and HCoV-NL63 (PDB:5N4K). A total of 73 compounds revealed binding energies below -9 kcal/mol to all investigated coronavirus NPs. The Venn diagram in Figure 2 shows the number of binding compounds to each of the single coronavirus NTD and the intersections between each other. The intersection in the middle shows the common 73 compounds binding to all NTDs with a binding affinity below −9 kcal/mol.

**Figure 3:**
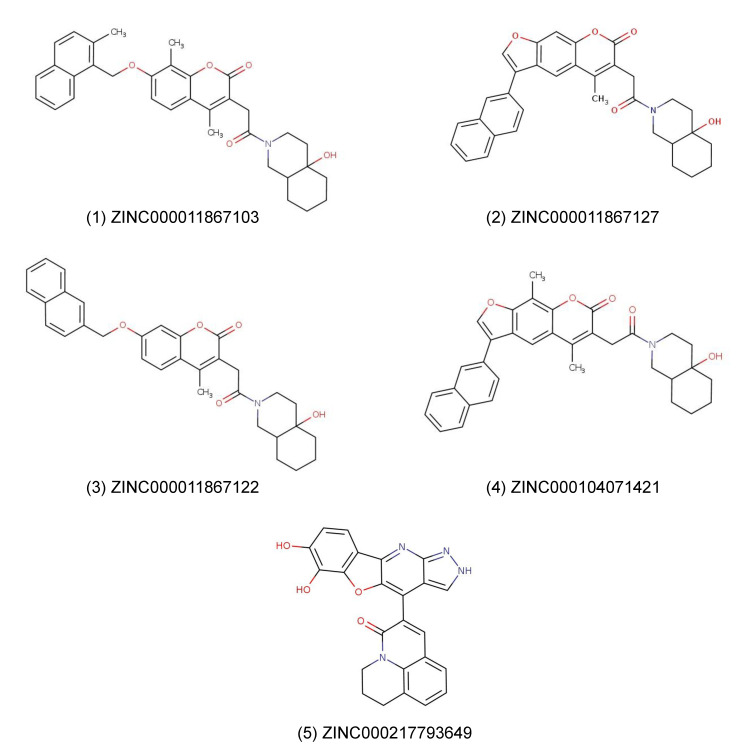
Chemical structures of selected candidate molecules from virtual docking used for subsequent in vitro studies.

From the results of this virtual screening, we selected five compounds for further investigations *(*Figure 3*)*. Table 2 displays the compounds with their different properties.

### 2.4. Microscale Thermophoresis

**Figure 4:**
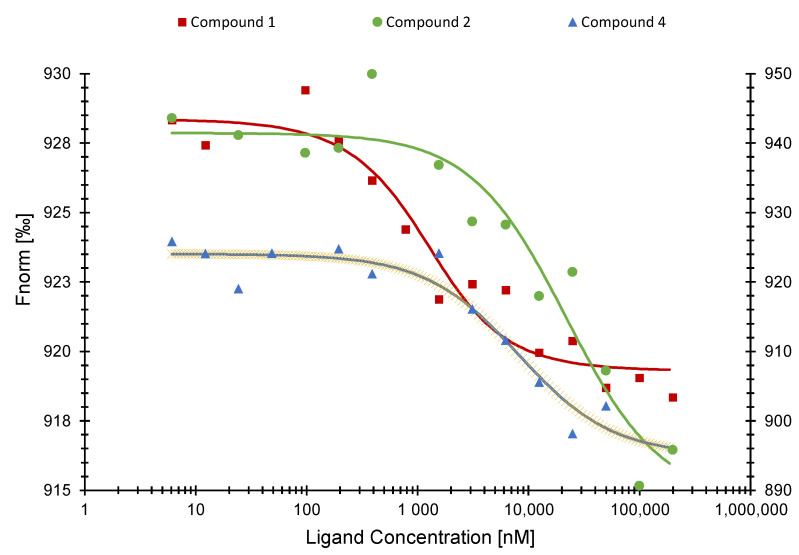
Binding curves of SARS-CoV-2 NP with compounds (**1**) (**left axis**), (**2**), and (**4**) (**right axis**) as determined by microscale thermophoresis.

By using microscale thermophoresis (MST) as a biochemical binding assay, we observed binding between the SARS-CoV-2 NP and compounds (**1**), (**2**), and (**4**). Figure 4 displays the binding curves of the individual compounds. The Kd values were 798 ± 2.03 nM (for compound (**1**)), 22.79 ± 1.52 µM (for compound (**2**)), and 8.07 ± 0.36 µM (for compound **4**), respectively.

**Figure 5:**
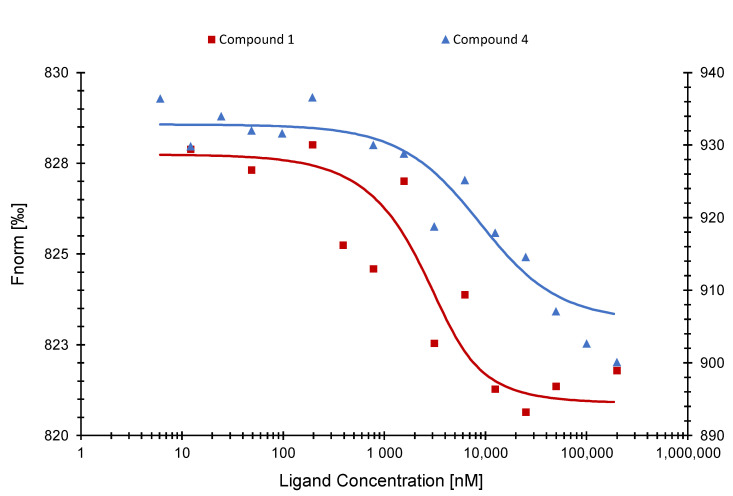
Binding curves of SARS-CoV-2 NP NTD with compounds (**1**) (**left axis**) and (**4**) (**right axis**) as determined by microscale thermophoresis.

We then studied the compounds (**1**), (**2**), and (**4**) using MST with SARS-CoV-2 NP NTD. Compounds (**1**) and (**4**) were bound as displayed in Figure 5. Compound (**1**) revealed a Kd value of 953.34 ± 31.88 nM and compound (**4**) of 7.77 ± 6.39 µM.

**Figure 6:**
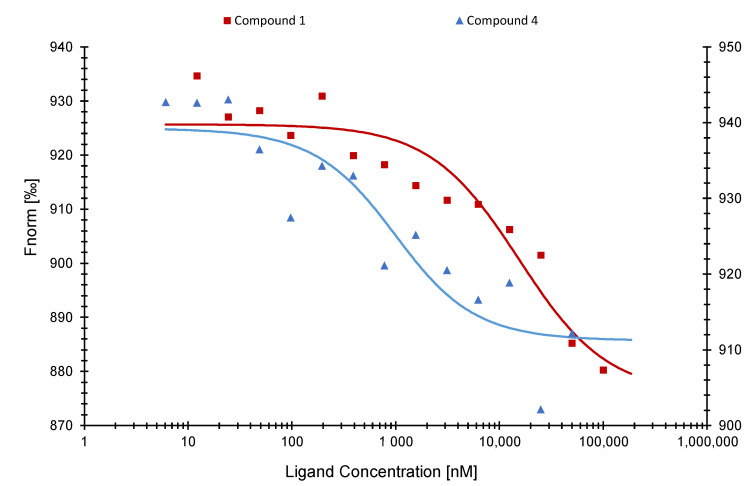
Binding curves of the SARS-CoV-1 NP NTD with compounds (**1**) (**left axis**) and (**4**) (**right axis**) as determined by microscale thermophoresis.

Next, we performed MST with compounds (**1**) and (**4**) with the SARS-CoV-1 NP. Compound (**1**) was bound to SARS-CoV-1 NP with a Kd value of 14.60 ± 0.44 µM. Compound (**4**) reached a Kd value of 674.02 ± 96.37 nM (Figure 6).

**Figure 7:**
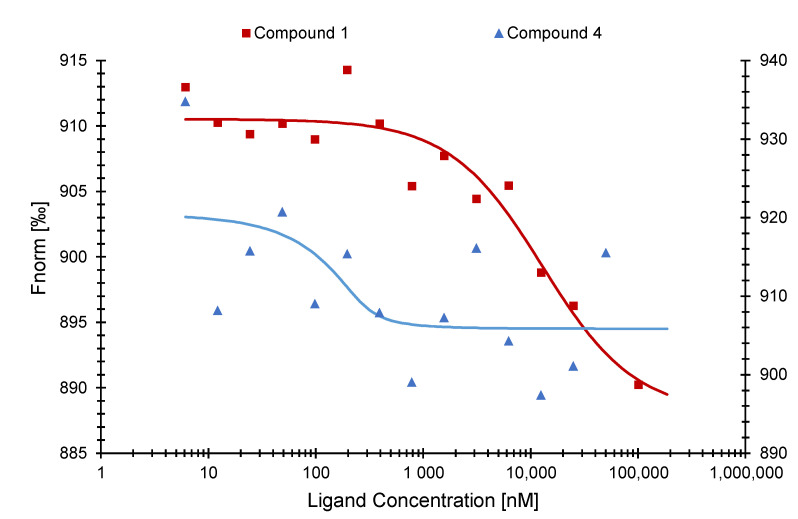
Binding curves of the SARS-CoV-2 NP omicron variant NP with compounds (**1**) (**left axis**) and (**4**) (**right axis**) as determined by microscale thermophoresis.

Finally, we tested compounds (**1**) and (**4**) with the omicron variant (B.1.1.529) of SARS-CoV-2 NP. Compound (**1**) resulted in a Kd value of 12.43 ± 0.26 µM. On the other hand, compound (**4**) was not bound to NP of the SARS-CoV-2 omicron variant (Figure 7).

### 2.5. Visualization of Ligand–Protein–Interaction

**Figure 8:**
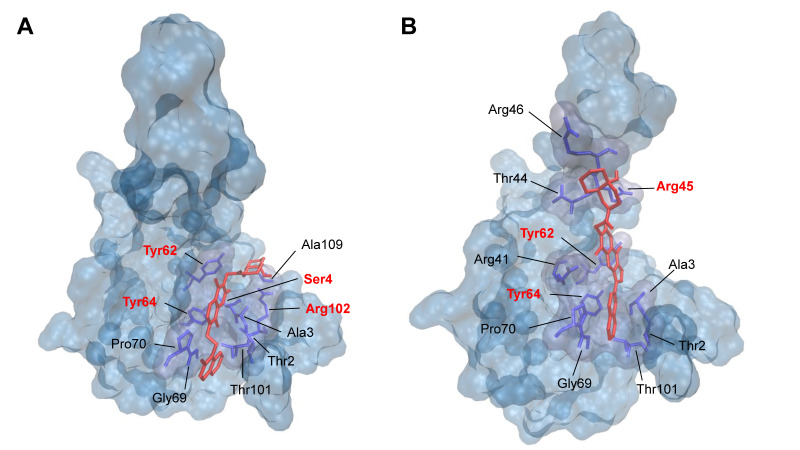
Molecular docking of (**A**) compound (**1**) and (**B**) compound (**4**) binding to SARS-CoV-2 NP NTD (PDB:6M3M). Red marked residues represent conserved amino acids in the sequence alignment.

To further study the interactions between active compounds (**1**) and (**4**) with the NP NTD, we performed molecular docking with AutoDock 4.2.6 and visualized the results by using VMD. Figure 8A shows compound (**1**) binding to the RNA-binding pocket of SARS-CoV-2 NTD (PDB:6M3M). The amino acids involved in this interaction were Thr2, Ala3, Ser4, Tyr62, Tyr64, Gly69, Pro70, Thr101, Arg102, and Ala109. Figure 8B displays the interaction of compound (**4**) with the residues Thr2, Ala3, Arg41, Thr44, Arg45, Arg46, Tyr62, Tyr64, Gly69, Pro70, and Thr101.

### 2.6. Cytotoxicity of Active Compounds towards A549 and MRC-5 Cell Lines

**Figure 9:**
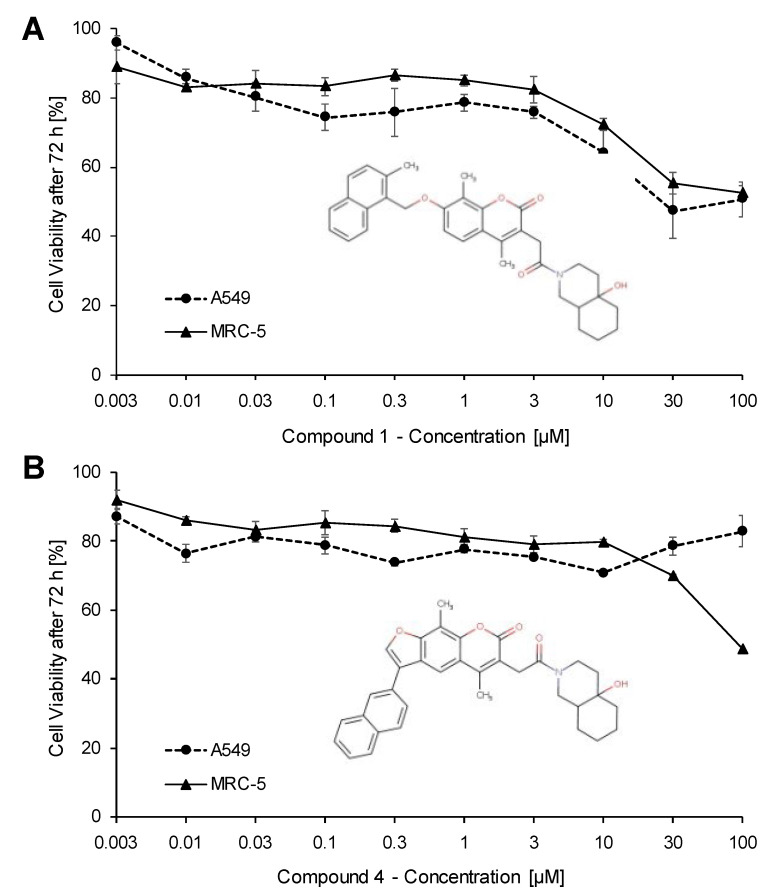
Cytotoxicity of compounds (**1**) (**A**) and (**4**) (**B**) towards A549 and MRC5 cells as determined by the resazurin assay. The dose-response curves represent the mean ± SD of three independent experi-ments for each cell line with each six parallel measurements.

Promising drug candidates should be non-toxic for human cells. Therefore, we tested compounds (**1**) and (**4**) using the resazurin assay and two different lung cell lines, A549 and MRC5. Figure 9 shows the cell viability after 72 h treatment with concentrations between 0.003 and 100 µM. Compound (**1**) resulted in IC_50_ values of 51.02 ± 8.13 µM for A549 cells and no toxicity for MRC-5 cells. Compound (**4**) resulted in an IC_50_ value of 93.39 ± < 4.34 µM for MRC-5 cells and no toxicity for A549 cells.

## 3. Discussion

**Figure 10:**
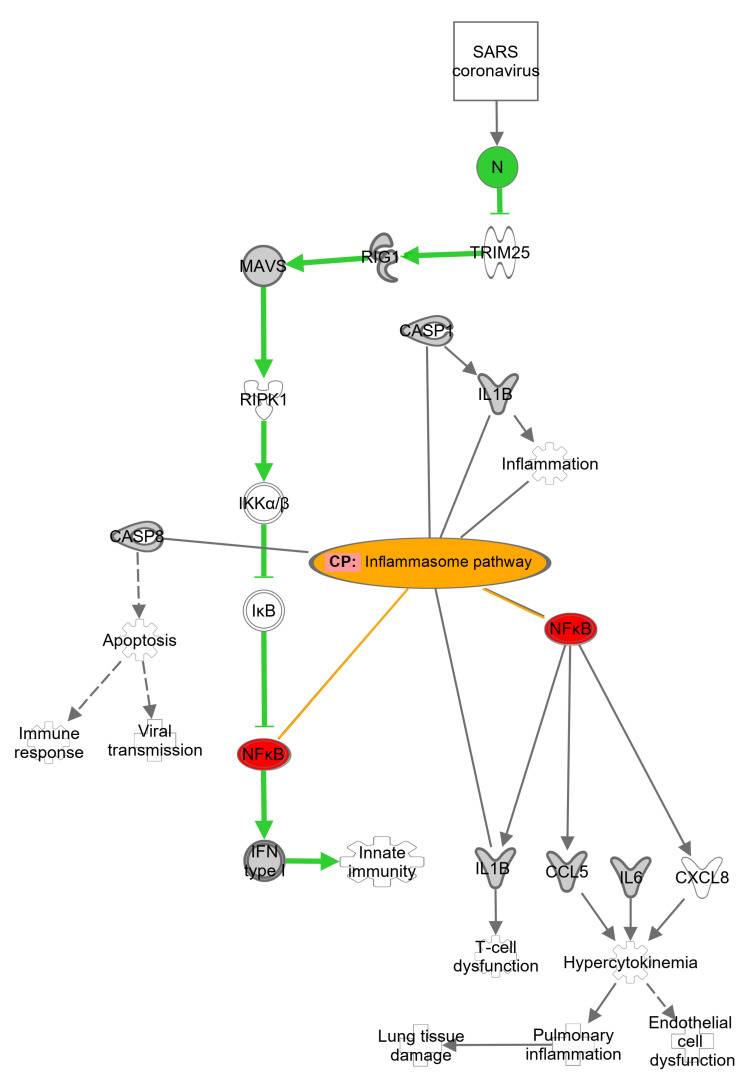
SARS-CoV-2-related signaling pathways in host cells. Green: NP-regulated pathway. Orange: Inflammasome pathway. Red: Connection of NF-κB to NP-regulated and inflammasome pathways.

The nucleocapsid protein plays a major role in SARS-CoV-2 infection. It interferes with the expression of the stress granule formation G3BP1/2 and RIGL1 receptor pathway genes [32,33], increases cytokine, and chemokine production [34], and interferes with many other pathways in the human body [35]. NP also interacts with the NLRP3 inflammasome in mice by boosting the assembly and activation. It increases proinflammatory reactions, such as multiplied expression of different interleukins (e.g., IL-1β, IL-6, TNF, etc.). Subsequently, the strong IL-1β expression stimulates the NF-κB signaling pathway, and even more cytokines are released. This can ultimately lead to a cytokine storm [20,21]. The correlation between NP, NLRP3 inflammasome, and NF-κB was also confirmed by using Ingenuity Pathway Analysis (IPA) (Figure 10). Therefore, NP is a considerable target for small molecules to fight acute SARS-CoV-2 infections and the subsequent long-term side effects termed “long-COVID”.

NP, as one of the main structural proteins of all coronaviruses (and many other viruses) should be more considered as an important drug target in addition to the coronaviral spike protein [4,13,36]. Some NP inhibitors of SARS-CoV-2 and MERS-CoV have been previously described [37,38]. However, most of all these studies reported solely in silico data [39,40,41,42]. A few examples of candidates investigated both in silico and in vitro were the synthetic drugs remdesivir and ceftriaxone. Remdesivir showed promising results but has to be further tested for safety and efficiency. Ceftriaxone, which is an antibacterial drug, demonstrated a high binding affinity to the SARS-CoV-2 NP NTD and is discussed as a potential drug against COVID-19 [40,41]. NP represents, therefore, not only an attractive drug target but also provides ample opportunities for natural product-derived compounds. Therefore, our goal was to find NP inhibitors by a combined in silico and in vitro approach.

As a first step, we studied the NP binding pocket by performing sequence alignments of the NTD domain of SARS-CoV-2, SARS-CoV-1, MERS-CoV, HCoV-OC43, HCoV-HKU1, HCoV-NL63, and HCoV-229E. SARS-CoV-1 showed the highest homology to SARS-CoV-2 with 92.54% similarity, followed by MERS-CoV with 60%. The key conserved residues that are important for the activity of NP were Ser4, Arg45, Arg60, Trp61 to Pro70, and Arg102. This result was also confirmed in the literature (Table 1).

The in silico compound screening of a ZINC-derived natural product library with 210,541 compounds resulted in 73 candidates that were bound to the NP NTDs of SARS-CoV-2, SARS-CoV1, MERS-CoV, HCoV-OC43, and HCoV-NL63 with free binding energies below -9 kcal/mol. We selected five of them according to lowest binding affinity, molecular weight, logP and commercial availability for subsequent in vitro experiments to confirm their binding activity. Four of them have similar structures with a coumarin core, except for differences in conformation and the position of OH- and methyl-groups. As a control, we performed docking using AutoDock4.2.6 with the known ligands, rapamycin, hydroxychloroquine, and ceftriaxone. The binding affinities were −7.75, −6.07, and −8.69 kcal/mol, respectively. The binding affinities were slightly higher in comparison to our compounds. Hence, the chosen compounds in this project demonstrated better in silico binding affinities to the SARS-CoV-2 NP NTD than the control drugs.

Microscale thermophoresis experiments with compounds (**1**), (**2**), and (**4**) indeed verified the in silico predicted binding activity. Compound (**1**) resulted in a Kd value of 798 ± 2.03 nM, which was the lowest of all active compounds, suggesting a good potential as SARS-CoV-2 NP inhibitor. On the other hand, compound (**2**) showed the highest Kd value with 22.79 ± 1.52 µM and was, therefore, excluded from further analysis. Next, we tested these active compounds with the SARS-CoV-2 NP NTD and confirmed that compounds (**1**) and (**4**) were specifically bound to the NTD. Even if the sequences of both NTDs are highly conserved, the conformation/folding of the NTD of SARS-CoV-1 may differ from the one of SARS-CoV-2 leading to different binding energies and Kd values [43].

The new omicron variants of SARS-CoV-2 that emerged at the end of the year 2021 [44] contained not only mutations in the spike protein but also in NP [45]. Therefore, we were interested to test our candidate compounds also with the NP of the SARS-CoV-2 omicron mutant. Compound (**1**) was bound with a Kd value of 12.43 ± 0.26 µM. This Kd value was higher than the one of compound (**1**) binding to the wildtype NP of SARS-CoV-2 (Kd = 798 ± 2.03 nM). The omicron variant has no mutations within the NTD sequence of NP (aa 48–174) but outside of it (B.1.1.529: P13L, ERS31-33del, R203K, G204R) (https://de.acrobiosystems.com/P4496-SARS-CoV-2-Nucleocapsid-protein-His-Tag-%28B11529Omicron%29.html (accessed on 16 May 2022)). These mutations can influence the conformation of the full-length protein, since they are present within the dynamic phosphorylateable linker region (LKR) [12,15] and, therefore, have an impact on the binding of compounds to NP. We also compared our MST results to the binding affinity of RNA to NP, since this is the natural ligand of this protein. Wu et. al. (2021) measured the binding affinities between NP and RNA via a fluorescence polarization assay and calculated a Kd value of 0.007 ± 0.001 µM for the NP wildtype. This value was slightly lower than the results of our active compounds for NP. This difference is possibly due to different methods used to determine the Kd value. These authors also found that the binding affinity to the NP NTD only was much lower compared to the full-length protein [46]. The Kd values for NP and NP NTD were similar for our compounds.

MST is a very sensitive method for analyzing the binding between proteins and ligands. We used the labeling MST technique, since the specific labeling of proteins with fluorescent markers lowers the disturbance of visible and UV-active ligands. This might also apply for the compounds displayed here because of their aromatic systems [47]. On the other hand, the high sensitivity might be a limitation of MST. It is crucial to work very precisely for sample preparation, since very small concentrations and volumes are used. Throughout the whole process, from labeling to measuring, there are many sources of error [48].

The molecular docking analyses suggested that compounds (**1**) and (**4**) interact with key residues of SARS-CoV-2 NP NTD, including Ser4, Arg45, Tyr62, Tyr64, and Arg102. This may explain the inhibitory capacity of the two compounds, since these amino acid residues are involved in the RNA-binding activity of NP.

Compound (**1**) and (**4**) showed low toxicity to human lung cells. Both compounds have a higher logP, making them more hydrophobic. This could possibly also affect their toxicity and off-target effects. Local anesthetics may serve as an example, since they have lipophilic characteristics [49]. However, it should be mentioned that hydrophobic characteristics can increase the cellular absorption because of higher affinities to the lipid membrane [50]. This could be a positive effect of these compounds.

Compared to the MST results, compound (**1**) reached Kd values of <1 for SARS-CoV-2 NP and NTD and a Kd value of <13 µM with the omicron variant, equal to the Kd value of SARS-CoV-1 NP. Compared to the IC_50_ value of A549 cells, the Kd values were more than 50-fold and 4-fold lower, respectively. For compound (**4**), a Kd value of ~8 µM was obtained for SARS-CoV-2 NP and NTD, and <1 µM for SARS-CoV-1 NP. The IC_50_ value occurring in MRC-5 was more than 11-fold lower. Hence, we concluded that compounds (**1**) and (**4**) did not show any cytotoxicity if used at a concentration range of the measured Kd values.

Compound (**1**) and (**4**) are derivates of the natural product coumarin. There was no further specific information available regarding their natural origin about both compounds. Chromene-derivates are usually secondary metabolites of plants such as Poaceae and Faboideae [50]. Typical plants containing coumarin are *Melilotus officinalis, Galium odoratum*, and *Prunus mahaleb*, but also plants from other families such as *Phoenix dactylifera, Dipterix odorata*, and several *Cinnamomum* species. Therefore, we have to leave it open whether our compounds are plant metabolites, metabolites in the human body, or semisynthetic derivatives that do not occur in nature. Nevertheless, we suggest that these two compounds may represent promising chemical scaffolds for further development against COVID-19 and other coronaviral infections.

## 4. Materials and Methods

### 4.1. Multiple Sequence Alignment

Multiple sequence alignment between NTDs of NP of 7 different coronaviruses, i.e., SARS-CoV-2 (PDB: P0DTC9), SARS-CoV-1 (PDB: P59595), MERS-CoV (PDB: K9N4V7), HCoV-OC43 (PDB: P33469), HCoV-NL63 (PDB: Q6Q1R8), HCoV-229E (PDB: P15130), and HCoV-HKU1 (PDB: Q5MQC6), was performed by using JalView (www.jalview.org (accessed on 28 June 2021)). Then, the web-service Tcoffee.crg.cat was selected [51]. The sequence homology was calculated using Clustal omega (https://www.ebi.ac.uk/Tools/msa/clustalo/ (accessed on 22 March 2022)).

### 4.2. Virtual Screening with Vina LC

Virtual screening and estimation of binding affinities was performed using a HPC “snakemake workflow” that implemented automatized steps of structural-based screening. The workflow uses Vina LC (version 1.3.0) for docking, Open Babel (version 3.0.0) for ligand energy minimization, and Biopython (version 1.75) for the preparation of the target structure. The library of 210,541 natural products was obtained from the ZINC database and screened with the main NTD of SARS-CoV-2 NP (PDB: 6M3M). The top 30% of results were displayed, and the cut-off value for rescreening with the NP NTDs of the other coronaviruses (SARS-CoV-1 PDB:2OFZ, MERS-CoV PDB:4UD1, HCoV-OC43 PDB:4J3K, HCoV-NL63 PDB:5N4K) was set to–9 kcal/mol.

### 4.3. Creating Grid Files and Molecular Docking with Autodock 4.2.6

Grid files of each NTD structure were created using AutoDock 4.2.6. The chosen NTD structures were obtained from the Protein Data Bank (www.pdb.org (accessed on 19 October 2021)): SARS-CoV-2 (PDB:6M3M), SARS-CoV-1 (PDB:2OFZ), MERS-CoV (PDB:4UD1), HCoV-OC43 (PDB:4J3K), and HCoV-NL63 (PDB:5N4K). The PDB file formats were transformed to PDBQT files (Protein Data Bank Partial Charge and Atom Type). Heterogenous atoms were removed. The grid box-dimensions (x, y, z) were fitted to include all possible active amino acids of the RNA-binding site and the structures were saved as a “gfp” format. AutoDock 4.2.6. A Lamarckian algorithm was used to perform docking between the active compounds and the SARS-CoV-2 NP NTD to identify amino acids responsible for hydrophobic interaction and H-bonds and furthermore to create the graphic presentation of the protein domain with the bound compound.

### 4.4. Recombinant Proteins

We used the recombinant SARS-CoV-2 nucleocapsid protein (biomol.com (accessed on 30 August 2021)), Cat.-No. PKSV030291), recombinant SARS-CoV-2 NP NTD (biomol.com (accessed on 30 August 2021 )), Cat.-No. KSR030538), SARS-CoV-2 nucleocapsid protein (B1.1.529 Omicron variant) (biomol.com (accessed on 11 April 2022)), Cat.-No. 101319), and recombinant SARS-CoV-1 nucleocapsid protein (bio-techne.com (accessed on 10 March 2022)), Cat.-No. 10710-CV-100).

### 4.5. Microscale Thermophoresis (MST) Analysis

Microscale thermophoresis was performed by using Monolith NT.115 (Nano Temper Technologies, Munich, Germany), as previously described [52]. Proteins were labelled using the Monolith Protein Labeling Kit RED-NHS 2nd Generation (NanoTemper Technologies, Cat.-No. M0-L011). The compounds were diluted in 16 different concentrations ranging from 6.1 nM to 200 µM, and labelled SARS-CoV-2 NP NTD (c = 3475 nM), labelled SARS-CoV-2 NP (c = 880 nM), labelled SARS-Cov-1 NP (c = 670 nM), or labelled SARS-CoV-2 omicron variant NP (c = 250 nM), respectively, were added 1:1. MST-Buffer (50 mM Tris-Base, 150 mM NaCl, 10 mM MgCl_2_ and 0.05% *v/v* Tween 20, filled up to 100 mL H_2_Odest.) was used for the dilution of the target proteins and the microscale thermophoresis preparations. The samples were incubated for 30 min in the dark and measured using the Monolith NT.115 instrument [53]. LED-power was set on 30% and MST-power on 10% for recombinant SARS-CoV-2 NP wildtype and omicron variant and SARS-CoV-1 NP, respectively. For recombinant SARS-CoV-2 NP NTD the LED-power was set on LED-power 40% and MST-power on 60%. Fitting curves with Kd values were calculated with MO.Affinity analysis software (NanoTemper Technologies, Munich, Germany).

### 4.6. Cell Lines

A549 lung cancer cells are frequently used in COVID-19 studies [33,54]. They were obtained from the Tumor Bank of the German Cancer Research Center (DFKZ, Heidelberg, Germany) and were maintained in Gibco™ RPMI 1640 medium with 10% fetal bovine serum (FBS), and 1% penicillin/steptomycin (PIS). Human diploid MRC-5 lung fibroblasts were kindly provided by Dr. Sebastian Zahnreich (Department of Radiation Oncology and Radiation Therapy, University Medical Centre of the Johannes Gutenberg University, Mainz, Germany). MRC-5 cells grew in Gibco™ DMEM, low glucose, pyruvate medium with 15% FBS, 1% PIS, and 1% Gibco™ MEM non-essential amino acids were used for cultivation. Both cell lines were incubated at 37 °C, 5% CO_2_, and 90% humidity. A549 cells were passaged every third day and MRC-5 cells every 6–7 days.

### 4.7. Cytotoxicity Assay

The cytotoxicity was tested by using a resazurin reduction assay [55,56]. Exponentially growing A549 and MRC-5 cells were seeded in 96-well plates at a density of 10^4^ cells/well. Different compound dilutions ranging between 0.003 and 100 µM were added in a total volume of 200 µL and incubated for 72 h. Thereafter, 20 µL/well resazurin (0.01% w/v) were added (Sigma Aldrich, Taufkirchen, Germany). Fluorescence was measured after 4 h incubation via an Infinite M200 Pro plate reader (Tecan, Crailsheim, Germany). Dose-response curves were generated by calculated the percentage of viable cells in treated samples compared to untreated control samples. The 50% inhibition concentration (IC_50_) was calculated from three independent experiments with six each parallel measurements.

## 5. Conclusions

In this work, we first performed in silico studies to find possible inhibitors of the nucleocapsid protein NTD of the seven currently existing human-pathogenic coronaviruses, especially SARS-CoV-2. From these results, we have chosen five compounds for in vitro testing using MST. The binding of compounds (**1**) and (**4**) to SARS-CoV-2 NP of the wildtype and NP NTD could be confirmed. Compound (**1**) was also bound to the NP of the omicron variant. Both compounds demonstrated low or no toxicity towards lung cells. Since it is one of the first attempts to find compounds against the coronaviral nucleocapsid protein, further improvements in compound selection and planning of future studies can be considered to emphasize the activity of these compounds. Despite the fact that compound (**1**) was bound alongside the SARS-CoV-2 and SARS-CoV-1 wild-type NP, the binding to the omicron variant was reduced. Compound (**4**) did not bind to the omicron variant. Therefore, it is desirable to enlarge the search for more compounds in the future and to conduct further experiments on the mechanism of action in addition to MST.

## Figures and Tables

**Table 1:** Amino acids involved in RNA binding of the NP NTD of different coronaviruses (SARS-CoV-2, SARS-CoV-1, MERS-CoV, HCoV-OC43, and HCoV-NL63). The sequence data were taken from UniProt.kb. Residues that occurred in more than one NTD are underlined.

NP NTD	Amino Acids Involved in RNA Binding	Reference
SARS-CoV-2	Asn1, Thr2, Ala3, Ser4, Phe5, Thr7, Ala8, Thr10, His12, Arg41, Arg42, Ala43, Arg45, Ile47, Arg48, Arg60, Tyr62, Tyr64, Arg102, Ans107, Tyr125	[10,12,13,15]
SARS-CoV-1	Lys14, Arg41, Arg45, Arg46, Arg48, Lys53, Arg60, Tyr62, Tyr64, Lys80, Arg102	[28]
MERS-CoV	Ser4, Trp5, Tyr6, Gly8, Tyr61, Tyr63, Arg100	[15,29]
HCoV-OC43	Gly8, Arg46, Lys50, Arg57, Tyr64, Tyr66, Arg104	[15,30]
HCoV-NL63	Ser4, Tyr6, Pro8, Gln43, Arg45, Arg47, Lys59, His61, Tyr63, Arg100, Lys105, Glu123	[15,31]

**Table 2:** Properties and docking results of compounds selected from virtual drug screening.

Nr.	Compound-ID	IUPAC	NTD	Binding Affinity [kcal/mol]	Molecular Weight [g/mol]	LogP
1	ZINC000011867103	3-[2-(4a-hydroxy-decahydroisoquinolin-2-yl)-2-oxoethyl]-4,8-dimethyl-7-[(2-methylnaphthalen-1-yl)methoxy]-2H-chromen-2-one	SARS-CoV-2 SARS-CoV-1 MERS-CoV HCoV-OC43 HCoV-NL63	−9.7 −9.8 −9.9 −9.4 −10.0	539.67	6.147
2	ZINC000011867127	6-[2-(4a-hydroxy-decahydroisoquinolin-2-yl)-2-oxoethyl]-5-methyl-3-(naphthalen-2-yl)-7H-furo [3,2-g]chromen-7-one	SARS-CoV-2 SARS-CoV-1 MERS-CoV HCoV-OC43 HCoV-NL63	−9.5 −11.3 −10.6 −10.2 −10.9	521.61	6.364
3	ZINC000011867122	3-[2-(4a-hydroxy-decahydroisoquinolin-2-yl)-2-oxoethyl]-4-methyl-7-[(naphthalen-2-yl)methoxy]-2H-chromen-2-one	SARS-CoV-2 SARS-CoV-1 MERS-CoV HCoV-OC43 HCoV-NL63	−9.5 −10.4 −9.7 −9.4 −10.1	511.62	5.530
4	ZINC000104071421	6-[2-(4a-hydroxy-decahydroisoquinolin-2-yl)-2-oxoethyl]-5,9-dimethyl-3-(naphthalen-2-yl)-7H-furo[3,2-g]chromen-7-one	SARS-CoV-2 SARS-CoV-1 MERS-CoV HCoV-OC43 HCoV-NL63	−9.7 −9.6 −9.5 −10.7 −10.6	535.64	6.672
5	ZINC000217793649	3-{5,6-dihydroxy-8-oxa-13,14,16-triazatetracyclo[7.7.0.0^2^,^7^.0^11^,^15^]hexadeca-1 (16),2 (7),3,5,9,11,14-heptaen-10-yl}-1-azatricyclo[7.3.1.0^5^,^13^]trideca-3,5,7,9 (13)-tetraen-2-one	SARS-CoV-2 SARS-CoV-1 MERS-CoV HCoV-OC43 HCoV-NL63	−9.3 −9.9 −9.5 −9.4 −9.4	424.42	4.197

## Data Availability

Data are contained within the article.
